# Supplementary material for: Development and real-life use assessment of a self-management smartphone application for patients with inflammatory arthritis. A user-centred step-by-step approach
Source: PLoS One. 2022 Sep 15;17(9):e0272235. doi: 10.1371/journal.pone.0272235 (PMC9477307; doi:10.1371/journal.pone.0272235)
Supplement: S1 File — (DOCX) [file pone.0272235.s001.docx]

**Supporting information 1. Qualitative studies.**

Appendix 1. Interview schedule. First qualitative study

Appendix 2. Data analysis and the consolidated criteria for reporting qualitative research (COREQ)

Appendix 3. Interview schedule. Second qualitative study

Appendix 4. Participants’ demographics and clinical features (n=42)

Appendix 5. Themes, sub-themes and quotes.

**Appendix 1. Interview schedule. First qualitative study**

1.Daily life with inflammatory arthritis.

Are you yourself playing the role of health manager or is there a family member who plays this role ? How ?

What are your disease’s manifestations in daily life ?

Do you have rituals for treatment intake ?

Did your lifestyle change since the disease onset ?

How do you solve practical issues with your treatment ?

Did you acquire skills on your disease and treatment ?

2. Practices of pharmacological and non-pharmacological treatments

How do you feel about you medication ?

Have you experimented medication changes ?

How do you imagine the way your medication works ?

What tricks did you find to control pain ?

3. The patient, his/her disease and social and work relationships

How the disease and treatment affect your body ?

What are your experiences with pain ?

How does your disease affect your professional and personal life ?

How do you interact with other people ?

**Appendix 2. Data analysis and the consolidated criteria for reporting qualitative research (COREQ)**

The interviews were audiotaped and verbatims were all transcribed. All data were de-identified to ensure confidentiality. Transcripts from each interview were analysed by using a thematic content analysis approach. They were read separately by the 2 researchers to identify preliminary themes. The interviews were further read to refine the preliminary themes. Any themes that were not covered by the preliminary codebook were added, then merged into main categories. Illustrative quotes of each group were selected. At each stage harmonisation was made between the 2 researchers.

COREQ: 32-item checklist

| **No** | **Item** | **Responses** |
| --- | --- | --- |
| **Domain 1: Research team and reflexivity** |  |  |
| Personal Characteristics |  |  |
| 1. | Interviewer/facilitator | GM, SG, FM |
| 2. | Credentials | GM and SG are MA, FM is PhD. |
| 3. | Occupation | GM and SG were employees of Unknows. FM was a PhD student. |
| 4. | Gender | Two males, one female |
| 5. | Experience and training | GM, SG, FM had conducted several qualitative studies in the field of health care, migration population, and politics |
| Relationship with participants |  |  |
| 6. | Relationship established | The researchers did not have prior relationships with participants |
| 7. | Participant knowledge of the interviewer | The participants expressed their informed non-opposition and knew the research objectives. |
| 8. | Interviewer characteristics | Employee of the digital company and interested qualitative studies in health care |
| **Domain 2: study design** |  |  |
| Theoretical framework |  |  |
| 9. | Methodological orientation and Theory | An ethnographic approach based on grounded theory was used. |
| Participant selection |  |  |
| 10. | Sampling | Selection was made by the rheumatologist on a mixed purposive-convenience basis |
| 11. | Method of approach | Telephone and email. |
| 12. | Sample size | 42 |
| 13. | Non-participation | The number of people who refused was not collected. We evaluated refusals to one quarter of all approached participants |
| Setting |  |  |
| 14. | Setting of data collection | The research took place at the participant's home and environment unless the participant wished otherwise |
| 15. | Presence of non-participants | No. Except a close one or relative if whished by the participant |
| 16. | Description of sample | Predetermined profiles as to their age, sex, socio-professional type of CIRD and DMARDs and number of previous DMARDs. |
| Data collection |  |  |
| 17. | Interview guide | Available upon request |
| 18. | Repeat interviews | No |
| 19. | Audio/visual recording | Audio-recording |
| 20. | Field notes | Field notes were made during and after the interviews |
| 21. | Duration | 60 min on average |
| 22. | Data saturation | Yes |
| 23. | Transcripts returned | No |
| **Domain 3: analysis and findings**z |  |  |
| Data analysis |  |  |
| 24. | Number of data coders | 2 in each study |
| 25. | Description of the coding tree | No |
| 26. | Derivation of themes | Themes were derived from the data |
| 27. | Software | No |
| 28. | Participant checking | No |
| Reporting |  |  |
| 29. | Quotations presented | Quotations are presented with identification of the participants |
| 30. | Data and findings consistent | Yes |
| 31. | Clarity of major themes | Yes |
| 32. | Clarity of minor themes | Not provided |

**Appendix 3. Interview schedule. Second qualitative study.**

1.History of patients pathway

Can you describe your disease course and possibly identify key landmarks?

Do you have associated co-morbidities ?

What DMARDs did you take ? What DMARDs are you currently taking? Who decided the treatment changes ?

In which circumstances were the prescriptions done ? How did you feel about it ?

2.Patient-rheumatologist relationship

Have you been followed by the same rheumatologist since the disease started ?How often do you see him/her? What is your relationship with him/her?

Can you discuss medical issues about with him/her?

Are there things you don’t tell your rheumatologist ?

Does your rheumatologist inquire how regularly you take your treatment ?

Have you mentioned side effects or aspects of the treatment you were worrying about ?

What are your expectations from your rheumatologist ?

Do you believe your rheumatologist has expectations from you ?

3.The treatment decision process

Is it always the same physician who prescribes your treatments? How did the decision-making process go with him/her?

Do you feel that your doctor has given you the keys to choose your treatment ? Did you doctor guide you in your choice?

How do you feel about this treatment compared to the previous ones ?

What was the most decisive in your choice: the discussion with the doctor? Did you consult relatives ? the internet ?

4.Daily medication management

How often do you take your medication?

How do you remember to take it? Do you have constrains with your treatment ?

Did you have any fears when you started the drug ?

What place does this treatment occupy in your daily life, in your projects?

What makes an oral or injectable treatment different for you: do you feel it works differently?

**Appendix 4. Participants’ demographics and clinical features (n=42)**

Qualitative studies were performed in May to June 2016 and March 2019 to February 2020.

The interviews lasted 60 min on average (range 45-120 min).

| Sex | Male | 9 |
| --- | --- | --- |
|  | Female | 33 |
| Age (median, range) |  | 51 (17-82) |
| Disease type | RA (mean age, 51) | 33 |
|  | SpA (mean age, 42) | 7 |
|  | PsA (mean age, 63) | 2 |
| Disease duration (years) | >10 | 22 |
|  | 4-10 | 7 |
|  | 2-4 | 9 |
|  | < 2 | 4 |
| Treatment | cDMARD* monotherapy | 11 |
|  | bDMARD** monotherapy | 10 |
|  | tsDMARD monotherapy | 9 |
|  | cDMARD+bDMARD combotherapy | 9 |
|  | cDMARD+tsDMARD combotherapy | 3 |
|  | Including 1^st^ or 2^nd^ line tsDMARD*** | 4 |
| **Socio-professional group**  **(French classification)** | Farmer | 1 |
|  | Craftsperson, merchant and company head | 1 |
|  | Senior managers, intermediate managers and intellectual professions | 11 |
|  | Intermediate professions | 5 |
|  | Employees | 10 |
|  | Manual worker | 1 |
|  | Retired | 7 |
|  | Others without professional activities | 6 |

*Conventional DMARD. ** biologic DMARD. *** targeted synthetic DMARD (JAK inhibitors)

RA, rheumatoid arthritis; SpA, spondyloarthritis; PsA, psoriatic arthritis

**Appendix 5. Themes, sub-themes and quotes.**

1. **Living with IA as a career**

| Description | Quotes |
| --- | --- |
| To search for causes, looking for information. Finding the right diagnosis and the right doctor | *“When I was diagnosed I read everything, I wanted to know everything” (Mrs P. Patient, RA.*  *“I was sure that it was that, when I saw the symptoms… Finally, I was happy to put a name to the disease” (Mrs D. Patient, RA).* |
| Patient–rheumatologist partnership | *“We are partners: we have to fight and to stop the disease.” (Mr. T. Patient, SpA). “Certolizumab had a good effect but not tremendous. We stopped after 18 months [...] Then we tried golimumab; it was an injection every 4 weeks and I said ‘be careful, it could be a bit long’ and it was a bit long indeed.” (Mr. T. Patient, SpA).* |
| Routines and habits | *“I found it difficult to tolerate the metho[trexate]: migraines, nausea, food intolerance, it made me exhausted the day I took it... and thus a trick consisted in taking it in the evening at bedtime [...] Now I take it on Saturday evening because it bothers me less.” (Mrs M. patient, RA)* |
| Side effects/disease complications. Handle situations in daily life; appropriate emergency responses | *“I had to have herniated disk surgery, I had to stop treatment. Surprise, surprise! you see that it's effective because you start to feel pain again....” (Mr G. Patient, RA). “It got really bad. I got a huge nail infection and had emergency surgery because I let it grow. I sent a picture to my rheumatologist.” (Mrs. D. Patient, RA).* |

1. **Patients’ skills and lay knowledge**

| Description | Quotes |
| --- | --- |
| Navigate the healthcare system, know which health professional to consult, when and how often, know what to expect from them. | *“Yeah, it takes a little time to even make an appointment with a doctor. And then what was really complicated was making an appointment at the hospital. I had called, I was in the middle of a crisis [...] and you can't get into the system [...] and I understood what I had to do, I can write, I can speak, I can be quite convincing and I think that someone less used to it than I am must feel really alone.” Mr. D, Patient, RA,* |
| Deal with information sources, know where to look for information, identify the most relevant sources. | *“We did blood typing because spondylitis is a lot of HLA B27... I'm not HLA B27 but it's the pelvic x-rays that showed sacroiliac fusion which is a hallmark of the disease, with inflammation syndrome always high and very high ESR... it's a marker for the disease plus very high C-reactive protein, it's the two markers that make it possible for it to be spondylitis.” Mr. S., Patient, SpA* |
| Know how to act with the rheumatologist, know what to say, not to hesitate to suggest changes | *“I have learned to talk to my doctors about my illness, to give the right information about the type, timing and spread of the pain.” Mrs B., Patient, RA* |
| Experimentation with the disease and treatment, drawing conclusions from these experiences. | *"My wife injects me every 8 days. Afterwards I manage myself, sometimes it's every 10 days. [...] I never reduce as I know the effects. On the other hand, I have had fun sometimes pushing at 10-12 days. Instead of doing it every 8 days, I gain 4 days because I feel good [...] [my rheumatologist] she doesn't like it. But I do my own thing.” (Mr. T. patient, SpA)* |
| Coping with illness and complications, dealing with common infections, managing minor treatment adjustments on your own initiative. | *“I have the maturity to recognize that... when I have 4 or 5 joints “coughing” at the same time I can self-medicate, and I take anti-inflammatory drugs to break the crisis and try to restart on my usual pain base...“ Mrs M., Patient, RA* |

1. **Patient treatment appropriation practices**

| Description | Quotes |
| --- | --- |
| The treatment ritual | *“Often I take it [methotrexate] and then… I sit down, I take a little 10 minute ritual even if the injection goes very fast. I need to concentrate a little bit and then, as it aches a little bit, I need to rest... sometimes I prefer to do it and then prepare my dinner... so I don't think when it aches ... but yes I like to be alone... (Mrs P. Patient, RA).* |
| Potential conflict between safety and adherence. | *“Once I hit my tibia with a chainsaw when gardening. We don't remember everything we were told and I did my injection however, so, I was lucky….., despite my 15 stitches I managed to heal well without it getting infected. [My rheumatologist] just reminded me that this kind of mistake should not be done and she was right!... It's just stupid, Etanercept has come into my life and it's a habit and well…., it's fine, but the chainsaw hit [...], I hit the jackpot”. (Mr G. Patient, RA)* |
| Effect of the daily mode of administration. | *“That's why, when I was offered baricitinib... I said to myself, ‘What is my risk?’. If it is beneficial for me, because ... the tocilizumab starts to decline, the result smells bad. I might gain 1 or 2 years when I will be fine, and then if it gets worse, we'll change [me and the rheumatologist]” (Mrs A. Patient, RA).* |
