# Supplementary material for: Development and real-life use assessment of a self-management smartphone application for patients with inflammatory arthritis. A user-centred step-by-step approach
Source: PLoS One. 2022 Sep 15;17(9):e0272235. doi: 10.1371/journal.pone.0272235 (PMC9477307; doi:10.1371/journal.pone.0272235)
Supplement: S2 File — (DOCX) [file pone.0272235.s002.docx]

**Supporting information 2. Hiboot Features and content examples**

Appendix 1. Hiboot screenshots

Appendix 2. Checklist

Appendix 3. Examples of helps in daily situations

Appendix 4. Information sources

Appendix 5. Examples of educational messages

Appendix 1. Hiboot screenshots


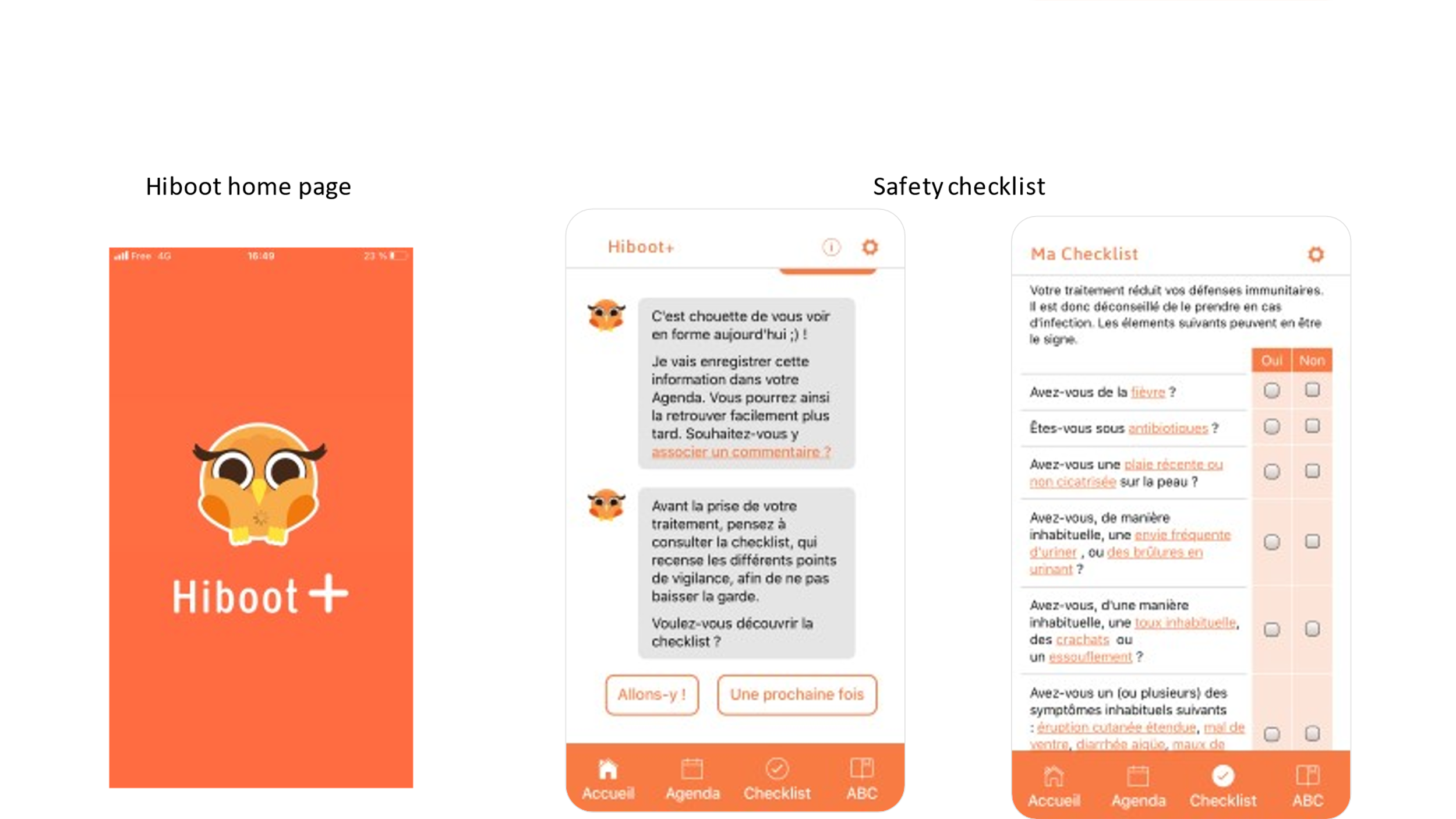


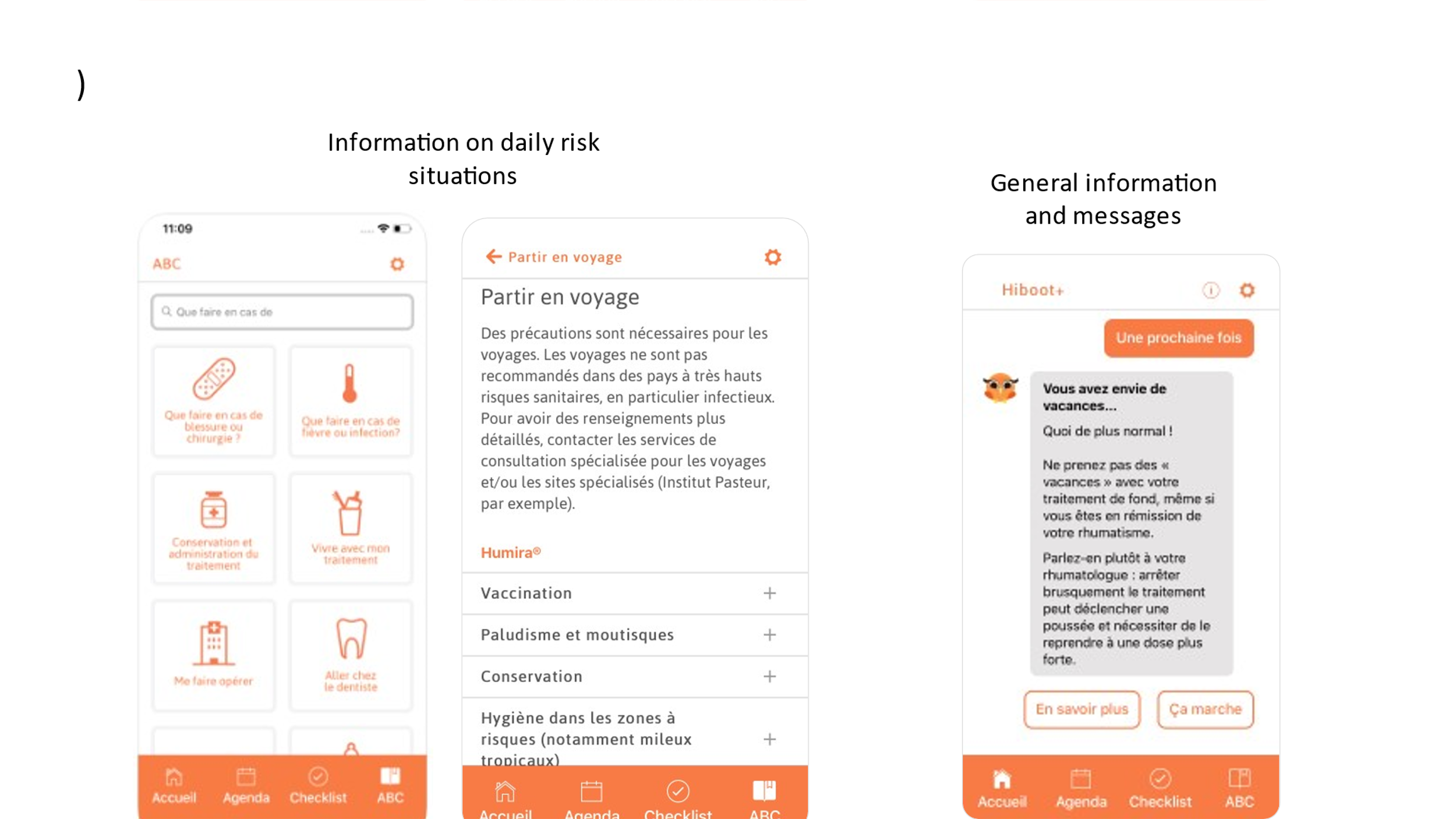


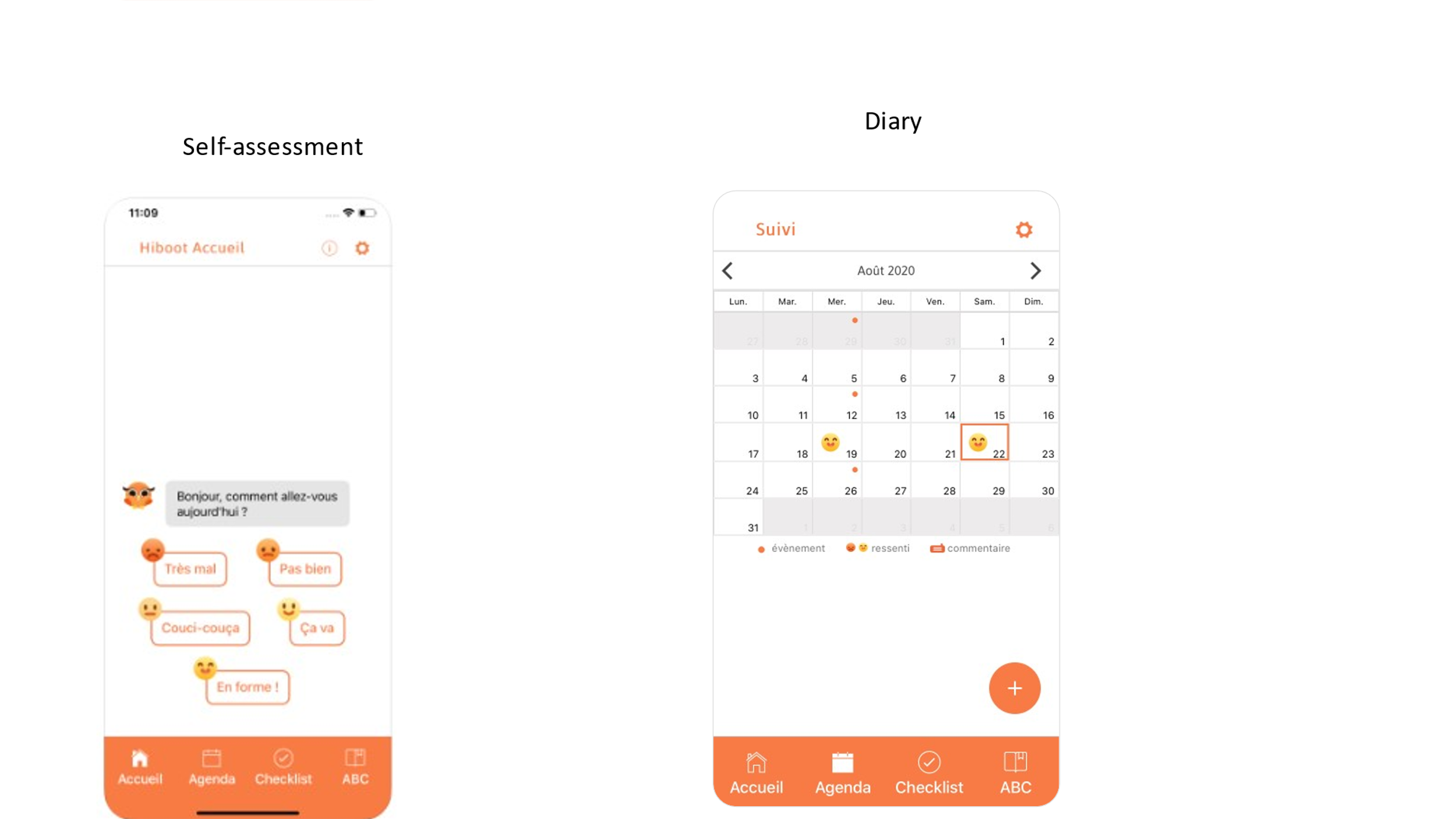


Appendix 2. Checklist.

| Do you have a fever? | yes | no |
| --- | --- | --- |
| Are you on antibiotics? | yes | no |
| Do you have a recent or unhealed wound on your skin? | yes | no |
| Do you have an unusual urge to urinate or burning while urinating? | yes | no |
| Do you have an unusual cough, sputum or shortness of breath? | yes | no |
| Do you have any of the following unusual symptoms: widespread skin rash, abdominal pain, acute diarrhoea, headache, sweating, severe loss of appetite? | yes | no |

If yes, you should not take your treatment. Consult your doctor.

If no, there is no contraindication for you to take your treatment

Appendix 3. Examples of helps in daily situations

Example: Gastroenteritis

| **General information** | Acute gastroenteritis is an inflammation of the mucous membrane of the digestive tract that causes diarrhoea. It may be associated with vomiting, abdominal pain and a moderate fever. It is most often viral and therefore highly contagious. In this case, it lasts less than 3 days. It can sometimes be due to a bacteria or a parasite. In this case, it requires specific treatments, such as antibiotics, and lasts about 2 weeks. In all cases, you should remember to stay well hydrated (drink plenty of water, broths) and wash your hands frequently to avoid transmitting your gastroenteritis. Moreover, if the symptoms persist, you should consult your doctor. |
| --- | --- |
| **Specific information** |  |
| Methotrexate | If you have a temperature equal to or higher than 38° and/or if you have chills, you should not take your *MED* and go to see your doctor quickly.  If your temperature is below 38°, it is not necessary to stop your *MED* treatment (non-severe gastroenteritis). |
| bDMARDs Subcutaneous (anti IL17 excluded) | It's time for your injection  In any case, you should not take your *MED*. If you have a temperature equal to or higher than 38° and/or if you have chills, go see your doctor quickly.  If your temperature is below 38°, you should wait until the end of the infection before taking your *MED*. If in doubt, contact your doctor.  If you have been treated with antibiotics, you should wait until the treatment is over before taking your *MED* again. |
|  | This is not the time for your injection  If you have a temperature equal to or higher than 38° and/or chills, you should see your doctor quickly.  If you are being treated with antibiotics, wait until the treatment is finished before taking your *MED*.  If you have a temperature of less than 38°, contact your doctor at the slightest doubt. If the symptoms persist or worsen, you should contact your doctor. In all cases, you should wait until the symptoms are over before taking your *MED* again. |
| Anti IL17 | It's time for your injection  In any case, you should not take your *MED*. If you have a temperature equal to or higher than 38° and/or if you have chills, go see your doctor quickly.  If your temperature is below 38°, you should wait until the end of the infection before taking your *MED*. If in doubt, contact your doctor.  If you have been treated with antibiotics, you should wait until the treatment is over before taking your *MED* again.  If you experience diarrhoea, unusual abdominal pain or blood in your stools while taking *MED*, you should be alerted to a possible inflammatory bowel disease. It is advisable not to inject *MED* and to consult your doctor. |
|  | This is not the time for your injection  If you have a temperature equal to or higher than 38° and/or chills, you should see your doctor quickly.  If you are being treated with antibiotics, wait until the treatment is finished before taking your *MED*.  If you have a temperature of less than 38°, contact your doctor at the slightest doubt. If the symptoms persist or worsen, you should contact your doctor. In all cases, you should wait until the symptoms are over before taking your *MED* again.  If you experience diarrhoea, unusual abdominal pain or blood in your stools while taking *MED*, you should be alerted to a possible inflammatory bowel disease. It is advisable not to inject *MED* and to consult your doctor. |
| bDMARDs Intravenous | It's time for your infusion.  If you have gastroenteritis at the time you receive your *MED* infusion, contact the department where you do your infusions to ask if the infusion needs to be postponed. |
|  | This is not the time for your infusion.  If you have a temperature equal to or higher than 38° or more and/or if you have chills, go quickly to see your doctor.  If you are being treated with antibiotics, it is advisable to wait until the treatment is finished before doing the infusion and to inform the hospital to postpone the infusion.  If in doubt, see your doctor. |
| Jak Inhibitors | In any case, you should not take your *MED*. If you have a temperature equal to or higher than 38° and/or if you have chills, go see your doctor quickly.  If your temperature is below 38°, you should wait until the end of the infection before taking your *MED*. If in doubt, contact your doctor.  If you have been treated with antibiotics, you should wait until the treatment is over before taking your *MED* again. |

*MED*: medication

Appendix 4. Information sources

Haute Autorité de Santé [French High Authority for Health], <https://www.has-sante.fr/> 07.15.2021),

Agence nationale du medicament (national drug agency, <https://ansm.sante.fr/>, 07.15.2021), National health service website (<https://www.ameli.fr/>, 07.15.2021)

Centre de référence sur les agents tératogènes (reference center for teratogens <https://www.lecrat.fr/>, 07.15.2021)

French Society of Rheumatology and the *Club Rhumatismes et Inflammations* (CRI). ([www.cri-net.com](http://www.cri-net.com), 07.15.2021) [24-28, 45]. The CRI is a scientific group of the French Society of Rheumatology created in 1997 dedicated to inflammatory and/or autoimmune diseases that includes academic experts (rheumatologists, internists, dermatologists and gastroenterologists) from France, Belgium and Italy. Within the CRI, a multidisciplinary committee is working on the development of practical guidelines for the use of DMARDs in everyday life for physicians. The guidelines have been published for more than 15 years in French and are recognized as a scientific reference for rheumatologists. Guidelines are issued by a process of literature review and expert consensus; they are regularly updated and presented on the CRI website in 3 categories: evidence-based medicine, official recommendations, expert opinions. Two active members of the CRI (TP, JS) were members of the Hiboot steering committee.

Appendix 5. Examples of educational messages.

.

| Flu: be one step ahead! Don't wait for the flu to become an epidemic, i.e. affect too many people: once vaccinated, you will need to wait 3 weeks to be immune to the virus.  Gain peace of mind for the winter by getting vaccinated in October-November. |
| --- |
| Feeling blue?  There are techniques to help you: massages, relaxation, not mentioning entertainment...  Here's some great information from Hiboot |
| Dairy-free diet: where to find the calcium?  Some patients are tempted to remove milk or dairy products from their diet with the idea of improving their arthritis, although the effectiveness of this type of diet has not been proven. If you are still inclined, talk to your doctor about replacing the lack of calcium by tablets or in calcium-containing mineral waters. |
| Do you wake up feeling like a robot?  A hot shower helps to stretch your joints and back so you can get started faster! |
