# Supplementary material for: Development and real-life use assessment of a self-management smartphone application for patients with inflammatory arthritis. A user-centred step-by-step approach
Source: PLoS One. 2022 Sep 15;17(9):e0272235. doi: 10.1371/journal.pone.0272235 (PMC9477307; doi:10.1371/journal.pone.0272235)

**Supporting information 3. Users’ tests**

Patients’ interviews lasted from 90 to 120 min and rheumatologist’s interviews lasted from 30 min to 1 hr.

Appendix 1. Demographics of patients and rheumatologists in the users’ tests September 2018

Appendix 2. Patients and rheumatologists quotes in the users’ tests September 2018

Appendix 3. Demographics of patients in the users’ tests in December 2019.

Appendix 4. Overlook of the work with the patients on the current version.

**Appendix 1. Demographics of patients and rheumatologists in the users’ tests September 2018**

**Patients**

| Patients No | Sex | Age (yrs) | Disease type | Disease duration (yrs) | App use |
| --- | --- | --- | --- | --- | --- |
| 1 | female | 48 | RA | 6 | Regular* |
| 2 | female | 50 | SpA | 19 | Regular* |
| 3 | male | 30 | SpA | 10 | Regular* |
| 4 | male | 67 | RA | 27 | Had dropped out |
| 5 | male | 26 | RA | 18 | Had dropped out |
| 6 | female | 47 | RA | 20 | Regular* |
| 7 | male | 33 | SpA | 8 | Occasional |

RA, rheumatoid arthritis; SpA, spondyloarthritis

*Regular use: at least once a month.

**Rheumatologist.**

| Rheumatologist No | Sex | Work location | Degree | App use recommendation |
| --- | --- | --- | --- | --- |
| 1 | male | University hospital | MD.PhD | No, never |
| 2 | male | Community hospital | MD. | No, rarely |
| 3 | female | Private office | MD. | Yes, regularly |

**Appendix 2. Patients and rheumatologists quotes in the users’ tests September 2018**

**Patients quotes.**

|  | Positive comments | Negative comments |
| --- | --- | --- |
| The features are appreciated but some titles (“monitoring” for self-assessment) are not well understood. | *"I like the owl, it's nice, it's cute."* | *" Monitoring" I don't really see the point of it."* |
| Navigation is not optimal |  | *"I feel like if I don't respond the recall is going to stay on hold."*  *"I used to do my injections every 4 weeks but if I feel I'm doing well I try each time to shift a few days to save one or two injections in a year. Now I do it every 5 weeks. But when I wanted to change on Hiboot, I had to do it all again..."* |
| Checklist.  The checklist utility is well understood but repetition is boring | *"Once I answered the checklist and the application told me to skip the injection. I wasn't expecting it, I was tired, I had a bit of a fever but I didn't think anything of it."*  *" There I put yes yes yes, but I think it still makes me ask the questions."* | *"It's a bit boring, it's always the same questions, it's six or seven questions in a row.*  *"Sometimes I do the checklist after the injection just to..."* |
| Situational aids  Situational aids are appreciated and reassuring. Participants would like more functionalities and better navigation to find the aids. | *"It goes straight to the point and that's the biggest advantage, it clears my doubts."*  *"What I read answered my questions."*  *"It is very reassuring for me to have answers. I appreciate the fact that I have a bit more support than with my rheumatologist who I see every 6 months. It's a bit like a cuddly toy that whenever I have questions I can ask him."* | *"And if I have to travel, there's the plane, the train, but there's no boat... And as I'm going on a cruise...*  *"I changed a dental crown, I had a small abscess, the dentist put me on antibiotics and I think there is one... which you shouldn't take with Methotrexate. I found out yesterday that it's specified in 'self-medication'. But it should be in dental care or dental abscesses instead."* |
| Reminders are appreciated but some participants use their smartphone reminder.  Difficulties in programming reminders. Be more flexible in the programming of recalls. | *"I think reminders are great!"* | *"I don't understand, I don't have a take on July 3rd. Tomorrow... I have nothing tomorrow. Oh yes, I have my « Metho ». It tells me I've got a take tomorrow but it doesn't tell me what..."*  *"I have another alarm to remember to take my folic acid but you know, sometimes with the holidays it's complicated... and it's not convenient to have both..."* |
| Counselling messages are appreciated | *"Hiboot advices yes I read them and I find them useful."* |  |
| Self-assessment/monitoring  The vertical format of the self-assessment is disturbing (first version), as users are used to a presentation closer to their smartphone diary.  Users express expectations of a calendar function. |  | *"It didn't speak to me so I skipped it."*  *"I would like to note in the follow-up "such and such a day I had this" with the date "*  *"I would have preferred something like a Google calendar with my day of intake. Something where I can project myself more day by day."*  *"What bothers me is that when I go to “follow up” you don't really like to wander around in it."* |

| Suggestions | *"It would be nice to be able to schedule one' s medication order a few days before.*  *“Why not add in hospital appointments."*  *"I'd also like a reminder for my folic acid."* |
| --- | --- |

**Rhumatologists’ quotes.**

| Themes | subthemes | Quotes |
| --- | --- | --- |
| Recommend the app | Requires a proactive approach. | *"I uninstall and reinstall the app in their presence in consultation to show them [the patients] how it works" R1* |
|  | Takes a great deal of time | *"That's [the app] one more thing to explain, we have so much to do..." R2* |
|  | Is not a routine practice. | *"I know it [the app] exists but I don't use it. It's not part of my routine when discussing with patients." R3* |
| Understand the scope of the application and its content | Identify the app in light of to the available information | *"It is a guarantee of independence from the pharmaceutical companies" R1*  *"We have too many different pieces of information, biosimilar booklets of 20 pages times 3..." R2.* |
|  | Know the content | *"I don't know exactly what's in it. I'm not sure what it is doing." R3*  *"When I explained the scope of the application, she finded it interesting and said she would download it" R2* |
| Identify which patients to recommend the app to | A reluctance in principle | *"Some people, even giving their phone number is complicated. R2* |
|  | Knowing whether patients use health apps | *"I don't know how much patients like this type of tools. R3. "Often patients don't use a lot of health applications" R2.* |
|  | A potential user profile identified as rather young | *"I talk about it to young people, a bit geeky, very active, not very organised" R1 "I don't talk about it to patients who are a bit older, not very smartphone users, very attached to the printed material" R1 "It's more for young people." R2.* |
|  | Doubts about the type of patients who would benefit from the app | *"Patients who are already very watchful, already anxious, will do their checklist etc. and this will "weigh down on their life experience". Whereas "the one who doesn't care, doesn't care." R3* |
| Feedback from patients | Positive | *"[a patient] it helped him tremendously" R1* |
|  | Negative | *She [a patient ]said she was not convinced by the app, that she "doesn't know what it adds". R2 She [a patient] believed it was for "calculating the disease activity", and for give "information on treatments". R2* |
| Content | Useful | *"The main point is the reminders" R1. "It is following the patients wherever they are in the world" R1* |
|  | Accessible | *"No problem with the handling of the app, it's quite nice". R1* |
|  | The checklist is not widely appreciated | *"The checklist is interesting at the beginning but it becomes redundant" R1* |
|  | The content is not adapted to all patients | *"It's not at the level of everyone." R2. "It is not applicable to all patients" R1* |

**Appendix 3. Demographics of patients in the users’ tests in December 2019.**

| Patients No | Sex | Age (yrs) | Disease type | DMARDs | Disease duration (yrs) | App use |
| --- | --- | --- | --- | --- | --- | --- |
| 1 | female | 45 | RA | Methotrexate SC | 7 | Yes |
| 2 | female | 40 | RA | Methotrexate oral | 7 | Yes |
| 3 | female | 45 | RA | JaK inhibitor | 20 | No |
| 4 | female | 55 | RA | JaK inhibitor | 15 | No |
| 5 | male | 35 | SpA | Etanercept | 10 | No |
| 6 | male | 50 | SpA | Etanercept | 2 | No |

The patients participated to the features, interface and navigation.

**Appendix 4. Overlook of the work with the patients on the current version.**


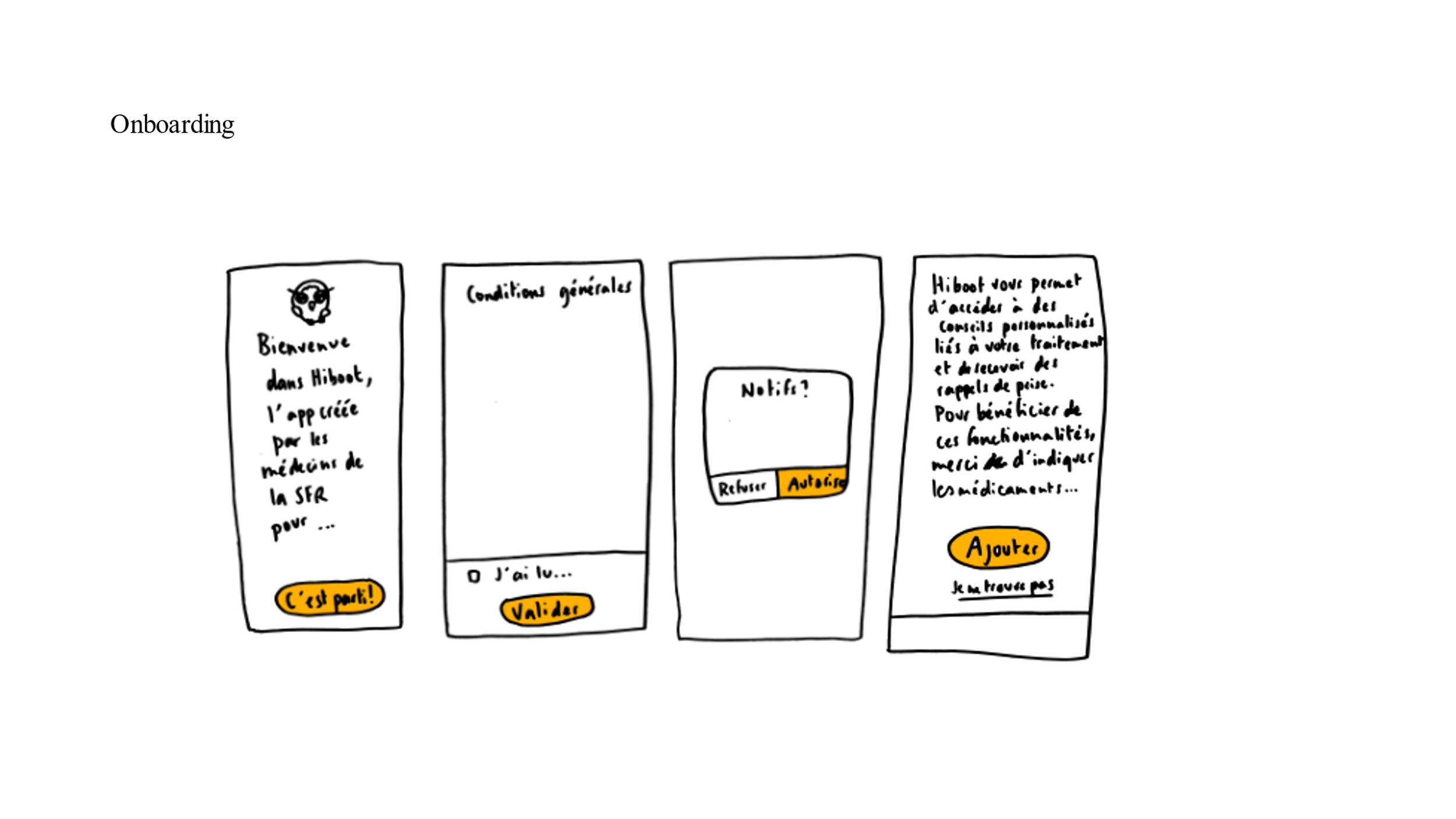


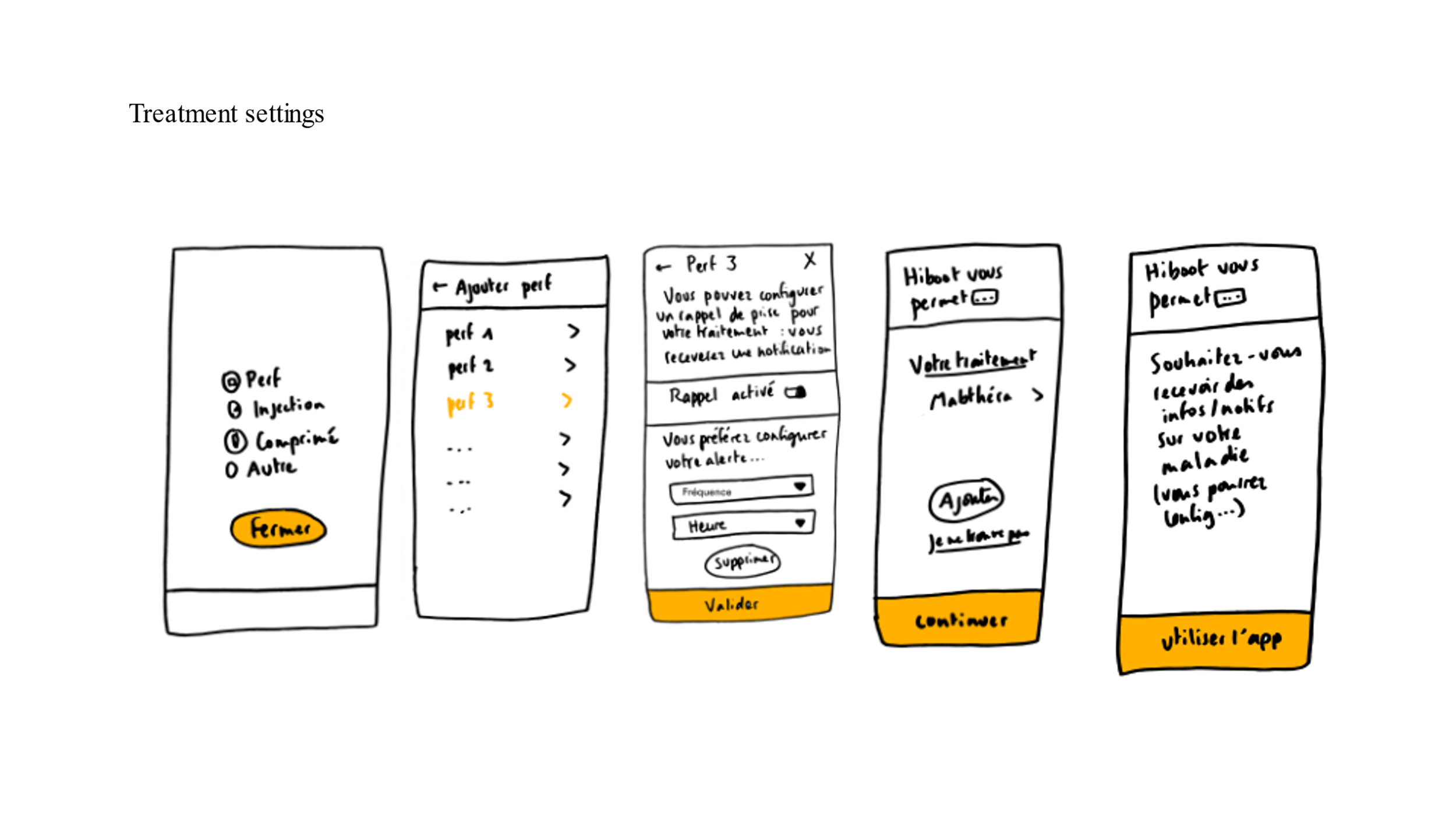


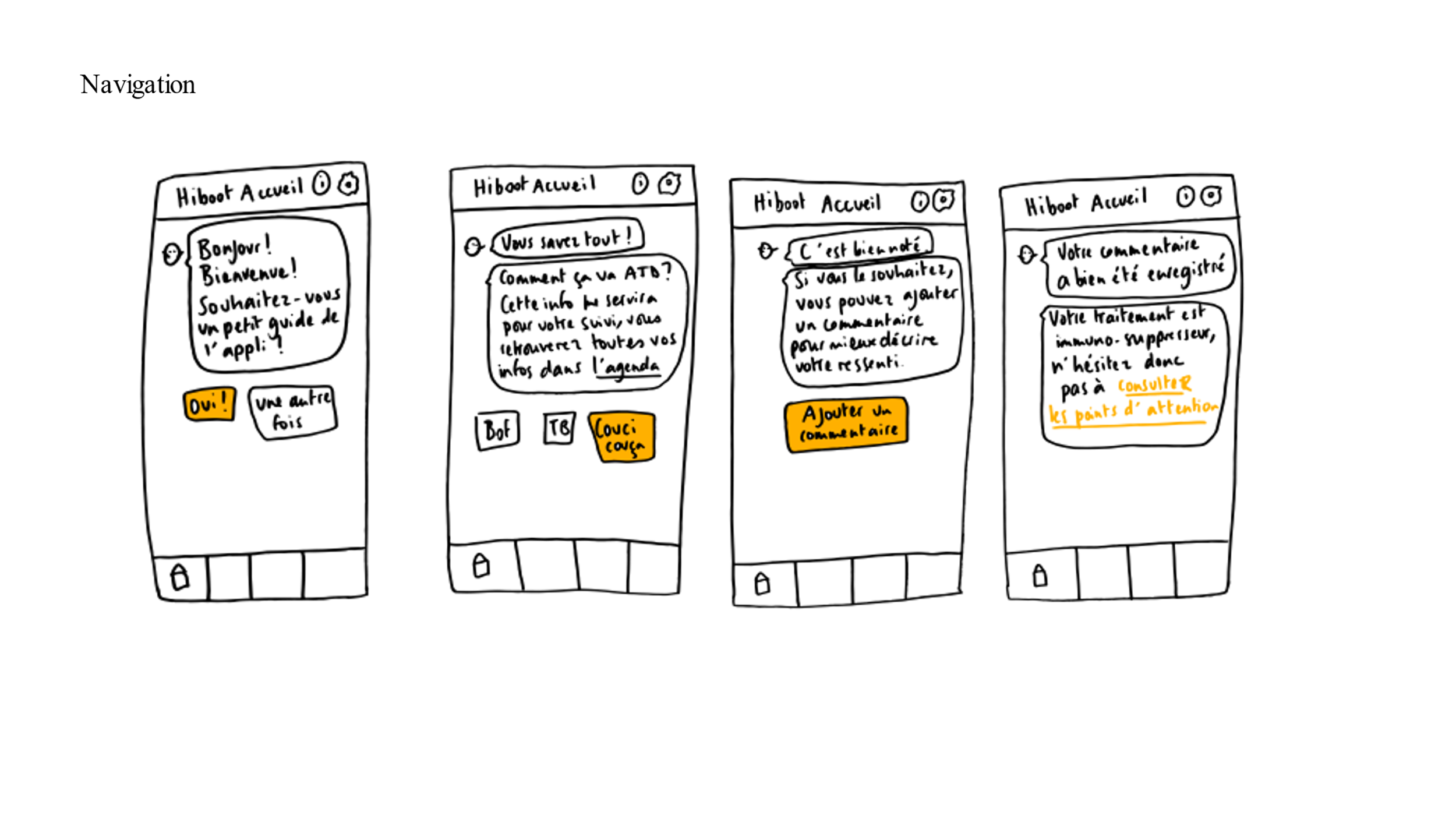


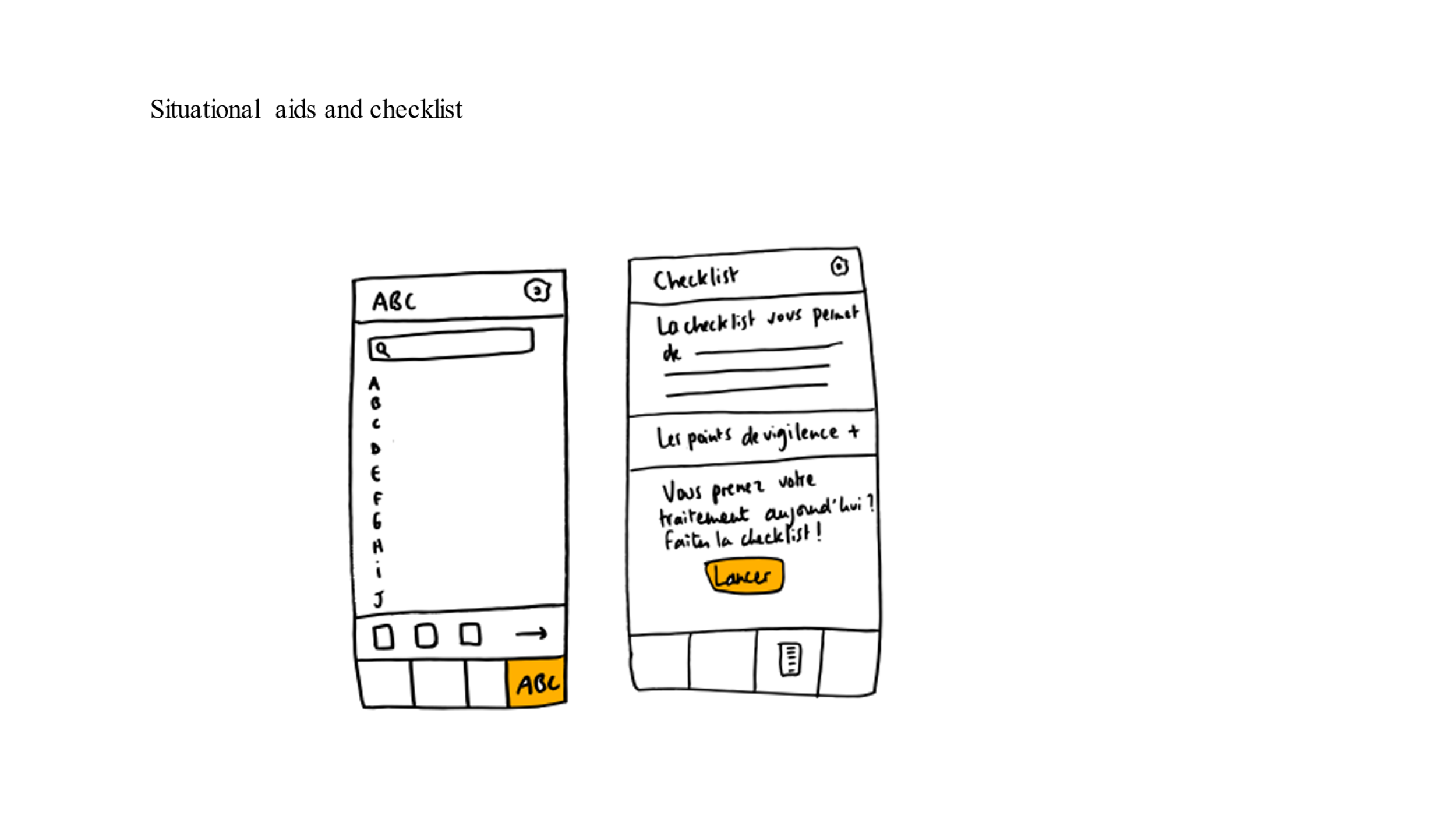

Supplement: S3 File — (DOCX) [file pone.0272235.s003.docx]
