# Supplementary material for: Development and real-life use assessment of a self-management smartphone application for patients with inflammatory arthritis. A user-centred step-by-step approach
Source: PLoS One. 2022 Sep 15;17(9):e0272235. doi: 10.1371/journal.pone.0272235 (PMC9477307; doi:10.1371/journal.pone.0272235)
Supplement: S4 File — (DOCX) [file pone.0272235.s004.docx]

**Supporting information 4. Number of views/users*/months from June to November 2020**

|  | Mean (SD) |
| --- | --- |
| Home page | 4.97 (0.24) |
| Diary | 3.18 (0.07) |
| Safety checklist | 3.97 (0.08) |
| Daily or at risk situations/symptom queries** | 3.38 (0.23) |
| Self-assessments | 2.61 (0.18) |
| Other treatment monitoring | 0.14 (0.01) |
| Medical appointments | 0.14 (0.02) |
| Treatment orders | 0.03 (0.004) |
| Other events/comments on diary | 0.09 (0.01) |

* user: individual whose app was in use once a month or more. ** these data are from the stores and are higher than in table 4, for which data were collected in Google analytics.
