# Supplementary material for: Development and real-life use assessment of a self-management smartphone application for patients with inflammatory arthritis. A user-centred step-by-step approach
Source: PLoS One. 2022 Sep 15;17(9):e0272235. doi: 10.1371/journal.pone.0272235 (PMC9477307; doi:10.1371/journal.pone.0272235)
Supplement: S5 File — (DOCX) [file pone.0272235.s005.docx]

**Supporting information 5. Scoring and comments at app stores**

Appendix 1: Scoring at app stores

Appendix 2. Comments at stores from 2018 to April 2021.

**Appendix 1: Scorings at app stores**


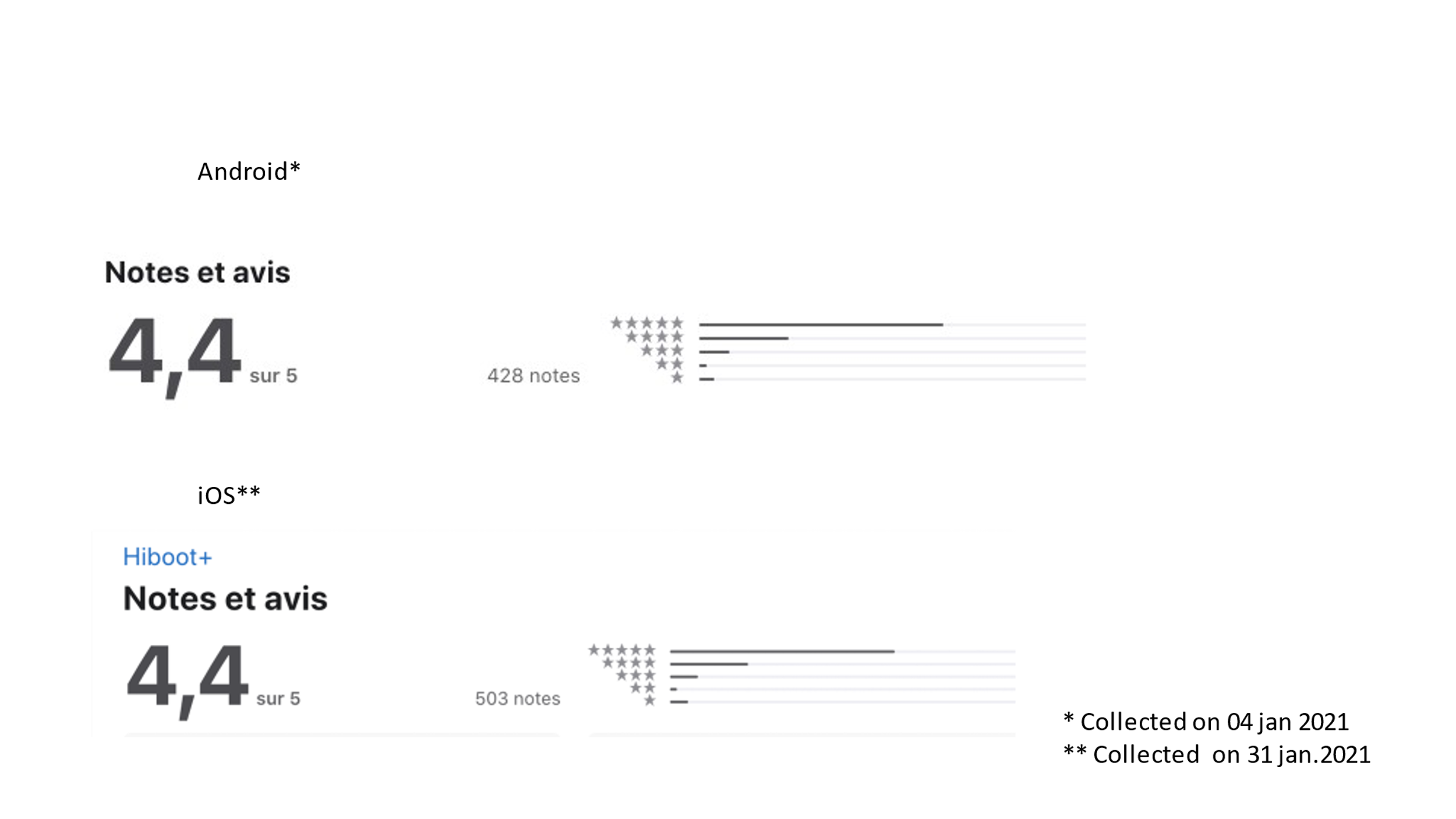


**Appendix 2. Comments on the stores from 2018 to April 2021.**

Total: 124. Overall positive: 47. Overall negative: 8. Bugs: 55. Missing functionalities:14

| Type of comments | N0 | Quotes in Google play | Number in Google play | Quotes in Apps stores | No in Apps store |
| --- | --- | --- | --- | --- | --- |
| Overall assessment | Positive  (34) | *“Pretty well thought out, especially the diary with the health status of the day and comments.”* | *23* | *“I like this app as it gives me lots of ideas and I manage my disease better”* | 11 |
|  |  | *“Very useful for reminders, practical and interesting information, very nice...”* |  | *“Clear and good advice”* |  |
|  |  | *“As a Hiboot user, I am happy to see this Hiboot+. The list of possible treatments has been extended. Quite a lot of improvements in the management of the frequency of treatments.”* |  | *“The idea of this app is good and I have been using it to follow my treatment for over a year.”* |  |
|  |  | *“Really happy that such an application exists for people who are careless with their treatment like me”* |  | *“This app helps me a lot in following my treatment. It's a great tool for reminders and pain monitoring... I highly recommend it!”* |  |
|  |  | *“Interesting easy to use app that guides me”* |  |  |  |
|  |  | *“Very satisfied with this app” “Great app”* |  |  |  |
|  | Negative  (7) | *“I confirm the other comments...Notifications do not appear at all. The old version worked perfectly Hiboot+ is a real disappointment on many aspects, no notifications, poorly thought out checklist”* | *4* | *“It's a pity because it was perfect before.”* | 3 |
|  |  |  |  | *“An update that removes the treatment is a shame. Also 4 weeks does not mean 1 month. I can't trust the treatment reminder which is the purpose of this app”* |  |
|  | Thanks (6) | *“Thank you for this update and for your work”* | *1* | *“A big THANKS to the team who set up this application and is running it.”* | 5 |
|  |  |  |  | *“Sweet the reminders are working. Thanks to you”* |  |
|  | Most appreciated (7) | *“Useful app especially for treatment reminders.”* | *3* | *“For its reminder, especially when the intake is once a week”* | 4 |
|  |  |  |  | *“Being able to add comments is a real +”.* |  |
|  |  |  |  | *“The new monitoring is much friendlier than the previous one.”* |  |
|  | Least appreciated (1) |  |  | *“The checklist is boring”* | 1 |
| Technical bugs | Bugs in general (8) | *“The last version worked perfectly unlike this one which does not have treatment reminders, in spite of the settings. I regret having removed the previous one.”* | *3* | *“The update introduced a lot of bugs and even some basic functions don't work anymore (tracking, feedback...). Quick, fix it! “* | 5 |
|  |  | *“App less fluent and less functional than the former version”* |  | *“It's a real shame that an application that worked very well and had excellent ergonomics, is so broken since the last update.”* |  |
|  |  |  |  | *“I lost all my data that I had to re-enter, and I have no more alerts!”* |  |
|  | Intervals (11) | *“For the basic treatment reminders, there is no option to enter the number of days “* | *6* | *“My treatment must be taken every fortnight, how can I change the 14 days to 15 please?”* | 5 |
|  |  | *“And it is not possible to register the vitamin D intake every three months.*  *It's a pity that other medications cannot be registered when they are taken occasionally.”* |  | *“I have 2 injections of Etanercept per week so I can't set this up”* |  |
|  | Checklist (10) |  |  | *“Since the last update, it is no longer possible to do your checklist. Of course the interface is nicer, but the application does not fulfil its primary role anymore, it's a crying shame!”* | 10 |
|  |  |  |  | *“Like everyone else, since the last update, impossible to do the checklist”* |  |
|  | Reminders (19) | *“Everything was working fine before but for several months now nothing, it's really annoying. I used it a lot for that very reason.”* | *15* | *“I take my treatment every 12 days and now the reminders are day after day.”* | 4 |
|  |  | *“As for the other users, no reminders.. It's a shame because I think the app could be good, but without this function its usefulness is reduced”* |  |  |  |
|  | Self-assessment (4) |  |  | *“Since the last update, impossible to enter my feedback.”* | 4 |
|  | Messages (2) | *“Interesting things but every time I only get the beginning of the message and if I click on it I never find it.* | *2* |  |  |
|  |  | *“Too bad I can't read the messages properly!”* |  |  |  |
|  | Not available (1) | *“Hello' the Hiboot application is not to be found on the play store, it used to be a few months ago.”* | *1* |  |  |
| Missing functionalities | My treatment is not in Hiboot (9) | *“Hello the hospital recommended this app to me in August. However, since then my treatment, Ixekizumab, has still not been added to the list, I can' t use the app if my treatment is not on board.”* | *4* | *“My medication is not in it, Ixekizumab, so the app is stuck on the front page, I can't use it. It's a bad thing that I can't use the features in the meantime, like the diary, the advices etc...”* | 5 |
|  |  |  |  | *“My rheumatologist gave me a leaflet and recommended this app! "The problem is that my treatment is not listed...what a pity!”* |  |
|  |  |  |  | *“I am on Tofacitinib, 2 tablets a day morning and evening. I would have liked to find it on the app… “* |  |
|  | Enter the dosages (1) | *“The application is not bad but it should be possible to enter the dosage of specific drugs which is not necessarily the same for each person...”* | *1* |  |  |
|  | Use the diary (1) |  |  | *“Impossible to note that I have done my injection on such and such a day”* | 1 |
|  | Print the diary (1) |  |  | *“Hello, could we have the option to print out the diaries with the feedback and comments so that we can give them to the doctor who is following us.”* | 1 |
|  | Export the diary (1) |  |  | *“Too bad we can't export the diaries”* | 1 |
|  | Confidentiality (1) | *“Is it possible to use a password connect by fingerprint to keep the information confidential?” ** | *1* |  |  |

*Information is confidential because the data are stored in the app and cannot be exported
