# Supplementary material for: Development and real-life use assessment of a self-management smartphone application for patients with inflammatory arthritis. A user-centred step-by-step approach
Source: PLoS One. 2022 Sep 15;17(9):e0272235. doi: 10.1371/journal.pone.0272235 (PMC9477307; doi:10.1371/journal.pone.0272235)
Supplement: Attachment — Submitted filename: RESPONSES to reviewer VF 03152022.docx [file pone.0272235.s007.docx]

RESPONSES TO REVIEWERS.

We thank the reviewers for their valuable comments on the revised manuscript. As reviewer #2 considered that we had responded adequately to the comments, here are our responses to reviewer #1

Reviewer #1:

 The authors have submitted the revised manuscript entitled “Development and real-life use assessment of a self-management smartphone application for patients with inflammatory arthritis. A user-centred step-by-step approach’, which is much improved compared to the original version. However, the manuscript still has some issues that need to be addressed by the authors as suggested below.
Major comments

1. The conclusion reported in the abstract and the main body of manuscript is more like a commercial and selling comments rather than a scientific remarks in line with the major findings of the research study.

Response 1. We thank the reviewer for this comment meaning that real life use of a health app only partially reflects its validity and does not presume its usefulness for patients. However, the conclusion of the abstract “Hiboot is a free self-management app for patients with IA developed by a step-by-step process including patients and health professionals. Further evaluation of the Hiboot benefit is needed. “ does not appear to contain any over-interpretation or commercial comment. We would like to underline that this app was developed by rheumatologists and patients organisations who did not receive any remuneration or fees from pharma companies or from the French Society of Rheumatology, which was the promotor of this study. The Rheumatologists developed the app as part of their work within their university hospital. Only the digital company was paid for the development. Moreover, as the app is free of charge, the study cannot be considered as an advertising.

The need for further research and the limitations have been underlined in the discussion and the conclusion.

In response to the reviewer’s comment, we have deleted the last sentence in the conclusion “ However, the still-increasing number of current users seems a surrogate marker of its utility. “ lines 550-551. In the abstract the sentence “ still increasing usage curve” was also deleted due to the limited number of words line 67.

2. The authors report that they used a parametric technique i.e. chi squared test (line 186, page 9), which is incorrect. The chi squatted test is a non-parametric test, please correct the text.

Response 2. To respond to the reviewer’s comment, the text has been modified as followed by the author Pr Florence Tubach lines 167-170 : “Associations between participants’ willingness to use an mhealth app (grouped in 2 classes: “totally and rather agree” vs “rather not and not at all agree”) and participants’ characteristics were calculated by using chi-square test or Fisher’s exact test (for categorical variables) and Wilcoxon rank test (for quantitative variables) according to the statistical distribution.

3. Results Section Summary of the key emerging themes (Lines 248-289). In this section the authors report only names of themes and sub-themes without any direct quotes of patients. The authors need to report some representative quotes for each theme in this section.

Response 3. We apologize because it was not clear enough that all the quotes corresponding to the themes and subthemes were in Appendix 3 in supporting information 1. Due to the length of the main manuscript, we chose to present the quotes in supplementary files. However, as suggested by the reviewer, short quotes were added in the text box 1. We added this information in the manuscript lines 229-230.

4. Please delete ‘eAppendix’ throughout the text because it is mentioned as either ‘multimedia’ or ‘supplementary material’. Please use only one term for the supplementary material and double check the content and number of each of the supplementary material file.

Supporting information. Appendix.

Responses 4. We changed the terms as suggested (Appendix. Supporting information) in the text and supporting information files.

Minor comments
ABSTRACT
5. Line 77: Could you please report any specific dates/months in the reported period i.e. 2017-2020.

Response 5. The information has been added : “ from October 2017 to September 2020” in the abstract line 66, in the methods line 212 and in figure 3

KEYWORDS
6. Line 83: It would be better Not to report an acronym i.e. DMARDs as a keyword but spell it out and report it as ‘Disease-modifying antirheumatic drugs (DMARDs)’

Response 6. This has been changed : “ ‘Disease-modifying antirheumatic drugs (DMARDs)’ line 72


INTRODUCTION
7. Line 110: Please be specific and report whose education (patients or users) and about what are you referring to the following sentence “Few apps for IA patients have targeted education [9,.10,.15].”

Response 7. Thank you for this comment. Education stands for patient education (added line 98). Few apps had targeted education: as mentioned above, “Most mhealth apps for IA described in the literature focus on symptom tracking, including disease activity, pain and fatigue [9-12], as well as physical activity [13.14], treatment reminders and exchange functionalities [15].” Lines 86-88

8. Line 126: Please delete 'Comments: OK'

Response 8. Done

9. Line 129: Please add 'app’ before the term ‘stores’.

Response 9.Done line 117 and in the text

METHODS
10. Line 142: Please check and change ‘private office’ to ‘private clinics/hospitals’ as appropriate.

Response 10. Referring to the French health system organization, we changed “private office” to “private practices” line 130

11. Line 149: The authors report using ‘the grounded theory model’. Could you please provide a reference/citation about the 'grounded theory model' used?

Response 11. Indeed the information was missing. The grounded theory was first described by Glaser and Strauss. (Glaser BG, Strauss AL. The discovery of grounded theory, strategies for qualitative research. New York: Aldine de Gruyter; 1967). Since then, a number of variants have emerged. As suggested, a reference has been added Charmaz K. Constructing Grounded Theory: A Practical Guide Through Qualitative Analysis. London: Sage, 2006 [33].

12. Line 152: Please report what age was considered as ‘adults’. You have mentioned in the side comments the age of ≥ 18 years.

Response 12. Yes, adults were aged ≥ 18 years. Added line 139

13. Lines 186-188: All these statistical tests are non-parametric tests. Please revise the statement and report the techniques used and the types of data analysed by the tests.

Response 13. The sentence has been modified as suggested by the reviewer, line 167-170. Please also look at the response to comment 2.

14. Line 197-200: Step 4 User tests: 6 patients recruited (current users and dropped out) in the 1st round (Sep 2018). Please report the number of current users and dropped outs users included in this stage.

Response 14. The users’ features are available in the results lines 406-407 and in appendix 1 in supporting information 3 : 4 were regulars users, one was an occasional user and 2 had dropped out.

15. Lines 203-205: In the 2nd round (Dec 2019): 6 patients were also recruited. Could you please report whether all of them were current users or included some dropped outs users. if so please, report their number in each category.

Response 15. The information is available in the results lines 406-407 and in appendix 3 in supporting information 3 : 2 were regulars users, 4 were non-users. Non-users were important in this step to test the features, interface and navigation of the app.

16. Lines 214: The authors report ‘…added 2 years after the app’s launch..”. Please report the months/year during which the 2nd qualitative study was undertaken so that a clear timeline is available for the readers.

Response 16. We agree that the timeline of the step-by-step approach can be hard to follow. The timeline is detailed in figure 1. The 2nd qualitative study was carried out from March 2019 to February 2020. As suggested, each step of the timeline was added in the text.

17. Line 235: The authors harnessed app users’ comments. Could you please how many unique users' comments were collected and analysed.

Response 17. The number of comments is available in the results line 448: 124 unique users’ comments were analysed.
18. Were your patients the same people in the first and second qualitative studies or different people in each study?

Response 18. The patients were different in the two qualitative studies as mentioned in Appendix 2, Supporting information 1

19. The authors did different studies over 4-5 years. Could you please sign post the research activities and timeline reported in figure 1 in the text.

Response 19. For each step, we added this information in the text. Please look at the response to comment 16.

RESULTS
20. Lines 240-244: The authors report that there were 21 patients each in study 1 and study 2 so the total patients become 42. However, the authors report female patients as 33/41, which should be 33/42.

Response 20. We apologize for this typo. It was corrected in the text : 33/42, lines 224-225. The correct information is available in the table in appendix 2 in supporting information 1.

21. Please correct the total of your patients (n=42) in the following sentence, which shows total 41 = (11+30) patients.

Response 21. This was corrected in the text : 11+31, line 224-225 . The correct information is available in the table in appendix 2 in supporting information 1.

22. Lines 244-246: The authors report that "Eleven received methotrexate

monotherapy and 30 bDMARDs or tsDMARDs monotherapy." So there were 11+30=41 patients. Please double check whether you had 42 or 41 total patients in the studies.

Response 22. We apologize for this. This was corrected in lines 224-225. The total number of patients was 42, as reported in the table in appendix 2 in supporting information 1

23. Lines 248- Section Summary of the key emerging themes. In this section the authors report only names of themes and sub-themes without any direct quotes of patients. The authors need to report some representative quotes for each theme in this section.

Response 23. Please look at the response to comment 3. We added short quotes in the text box 1 and left full quotes in appendix 3 in supporting information 1.

24. Line 383: keywords: Could you please signpost the readers which keywords and where they are reported in the manuscript.

Response 24. We thank the reviewer for pointing that we did not give examples of keywords. Due to the length of the manuscript, an example was added in appendix 3, supporting information 2 and mentioned in the text lines 362-363.

25. Lines 480: Please double check whether the following is correct: views/month/users. It could be views/month/user. (Such as XX views per month per user (NOT users)).

Response 25. This was corrected lines 441-443

26. Lines 285-590: Please report what numbers given in parenthesis in these sentences show? These number could be confused and read as reference / citations.

Response 26 : this was corrected line 450-456.

DISCUSSION
27. Line 500: the authors report ‘…..4300 users so far.’ Could you please the exact date on which this number was ascertained?

Response 27. This was added line 466

28. Line 501: Preliminary studies: Please double check whether you mean Preliminary studies or previous/earlier studies. The later seems more appropriate in the context of the sentence. Please check whether this needs to be changed elsewhere in the manuscript.

Response 28. Indeed, this was not clear enough. The term “preliminary studies” stands for studies conducted in step 1. This was defined in lines 176 and 467.

29. Line 518: The authors report that “To our knowledge, no app of this nature is currently available for adult patients with IA.”. Again, in lines 551-552, the authors talk about other apps for patients with IA and state “However, this number seems substantial as compared with other apps for people with IA”. Please double check the contradictory statements and revise as appropriate.

Response 29. Thank you for pointing out this discrepancy. To our knowledge, no app of this nature is currently available for adult patients with IA because no other app is devoted to self-management particularly on medication management. As mentioned in the introduction, “Other apps for IA focus on symptom tracking, including disease activity, pain and fatigue [9-12], as well as physical activity [13.14], treatment reminders and exchange functionalities [15]. In line 551-552 we refer to apps dedicated to physical activity.”

This was clarified as follows “To our knowledge, no self-management app of this nature is currently available for adult patients with IA, particularly with respect to medication management. “ lines 483-484

“However, this number seems substantial as compared with other apps for people with IA dedicated to physical activity ”. line 517.
CONCLUSION
30. The conclusion is more like a commercial and selling statement rather than scientific remarks about the app and the study findings.

Response 30. Please look at the response to comment 1

Tables and boxes etc.
31. Text box 1 should include a column where the authors should report direct quotes from the participants.

Response 31. This has been done

32. Figure 3 caption, Please reports the dates month/years in the caption which only includes ‘since October 2017’ which should be ‘October 2017-Month/Year) to show the period a.

Response 32. This has been done

Reviewer #2: The adequately addressed my comments . They explained more in details the methodology and reviewed the language.

Response. We thank the reviewer for having considered our responses.
